# Influence of low concentration acid treatment on lithium disilicate core/veneer ceramic bond strength

**DOI:** 10.4317/jced.51081

**Published:** 2013-10-01

**Authors:** Hugo A. Vidotti, Rudan P. Garcia, Paulo CR. Conti, Jefferson R. Pereira, Accácio Ld. Valle

**Affiliations:** 1 Ph.D. Candidate. Department of Prosthodontics, Bauru School of Dentistry, University of São Paulo, Bauru, SP, Brazil; 2 Undergraduate Student. Bauru School of Dentistry, University of São Paulo, Bauru, SP, Brazil; 3 Professor. Department of Prosthodontics, Bauru School of Dentistry, University of São Paulo, Bauru, SP, Brazil; 4 Professor, Department of Prosthodontics, School of Dentistry, University of Southern Santa Catarina

## Abstract

Objective: This study evaluated the influence of low concentration acid treatment on the shear bond strength between lithium disilicate (LD) infrastructure and veneering porcelain. The surface morphology characteristic after this acid treatment was also examined. Study Design: LD reinforced ceramic cylinders (n=10) (IPS e.max Press, Ivoclar-Vivadent, Schaan, Liechtenstein) were treated (LD-treated) with a low concentration acid solution (Invex Liquid – Ivoclar-Vivadent, Schaan, Liechtenstein) or not treated with the acid solution (LD-untreated). They were veneered with a glass ceramic (IPS e.max Ceram, Ivoclar-Vivadent, Schaan, Liechtenstein). A metal ceramic group (CoCr) was tested as control. Shear bond strength (SBS) was conducted using a universal testing machine at 0.5 mm/min. Surface morphology characteristics after acid treatment were analyzed using scanning electron microscopy. Results: The acid treatment at low concentrations did not influence the SBS of the LD/veneering porcelain interface. The CoCr group showed the significant higher SBS value (35.59 ± 5.97 MPa), followed by LD-untreated group (27.76 ± 3.59 MPa) and LD-treated (27.02 ± 4.79 MPa). The fracture modes were predominantly adhesive for CoCr group and cohesive within the infrastructure for DL groups. Scanning Electron Microscopy (SEM) analysis showed no morphological differences between treated and untreated LD surfaces. Conclusions: Low concentration acid treatment did not improved SBS of veneering ceramic to LD and did not cause morphological changes on the LD surface.

** Key words:**Lithium disilicate, glass ceramics, acid etching, shear bond strength, scanning electron microscopy.

## Introduction

A great development of all ceramic systems for dental restoration occurred in the last 20 years, which provided to the clinician a more aesthetic alternative for anterior and posterior cases (1,2). All ceramic crowns have the potential to be more aesthetics than metal-ceramic restorations, since these metallic infrastructures cause an opaque appearance and metal borders may be visible in some situations.

The use of ceramics to manufacture dental crowns has evolved since the beginning of 20th Century. First crowns fabricated in pure porcelain in this period showed low clinical performance, besides they were very difficult to manufacture. With the evolution of metal alloy casting technics and the knowledge about mechanical behavior and bonding of porcelains fused to metal, starting in 50s, there was a great development in the production of total crowns and fixed prosthodontics that combine these two materials (3,4). In the same way, crowns made fully in ceramic material can be fabricated using ceramic reinforced structures, as alumina infiltrate by glass, glass-ceramic reinforced by lithium disilicate, densely sintered aluminum oxide or yttria-stabilized tetragonal zirconia veneered with their respective porcelain of in order to achieve aesthetics characteristics (5).

The system IPS Emax Press (Ivoclar-Vivadent, Schaan, Liechtenstein) is based on glass-ceramic reinforced by lithium disilicate crystals (60-65%). The material is injected in a mold of coating obtained by loss wax technic under high temperature and pressure. This system reduced the problem of contraction during the burn of ceramic, common in feldspathic materials due to the high pressure of injection in high temperature mold. Because of that, dimensional variation only occurs during the cooling, and it can be controlled by adequate expansion of the investment material (6).

A peculiar characteristic of reinforced ceramic by lithium disilicate, is the quality to be acid sensitive, in other words, it suffers morphological changes in front of acid treatment with hydrofluoric acid in different concentrations. This phenomenon occurs due to the micro-structural characteristics of the material. The main crystalline phase consists of elongated lithium disilicate crystals. The second crystalline phase consists of lithium orthophosphate. A glass matrix surrounds both crystalline phases. Hydrofluoric acid in 10% of concentration is capable to remove the glass matrix and the lithium orthophosphate crystalline phase exposing only lithium disilicate crystals, which create an irregular surface fundamental to a good adhesion (7-9).

Until the present time, it is not clear the effects of treatments using low concentration acids on the surface of lithium disilicate. The manufacturer recommends the use of an acid solution with approximately 0.6% of hydrofluoric acid and 1.7% of sulfuric acid by a period between 10 and 30 minutes, inside an ultrasonic container, after the divesting of ceramic pieces. This procedure would have the function of preventing problems in the bonding to the veneering porcelain. This protocol was followed in studies that analyzed lithium disilicate/veneering porcelain interface and high values of bond strength were found (10-12). However, no attempt was made to investigate the influence of it in the bond strength of this interface.

The present study evaluated the influence of low concentration acid treatment on the bond strength of lithium disilicate reinforced ceramics to veneering porcelain. It was also performed a surface morphology analysis by Scanning Electron Microscopy to characterize the morphological aspects of the surfaces after different acid treatments.

## Material and Methods

A lithium disilicate reinforced ceramic (IPS e.max Press, Ivoclar-Vivadent, Schaan, Liechtenstein) veneered with a glass ceramic (IPS e.max Ceram, Ivoclar-Vivadent, Schaan, Liechtenstein) was used in this study. Two experimental groups were made: LD-treated – lithium disilicate reinforced ceramic treated with acid solution of approximately 0.6% hydrofluoric acid and 1.7% sulfuric acid (Invex Liquid – Ivoclar-Vivadent, Schaan, Liechtenstein) after divesting and LD-untreated – lithium disilicate reinforced ceramic not treated with the acid solution. A metal-ceramic system (FitCast CoCr – Talladium, Valencia, EUA) veneered with IPS Inline porcelain (Ivoclar-Vivadent, Schaan, Liechtenstein) was used as control (CoCr). Chemical composition and properties of the materials tested are shown in  Table 1.

**Table 1:**
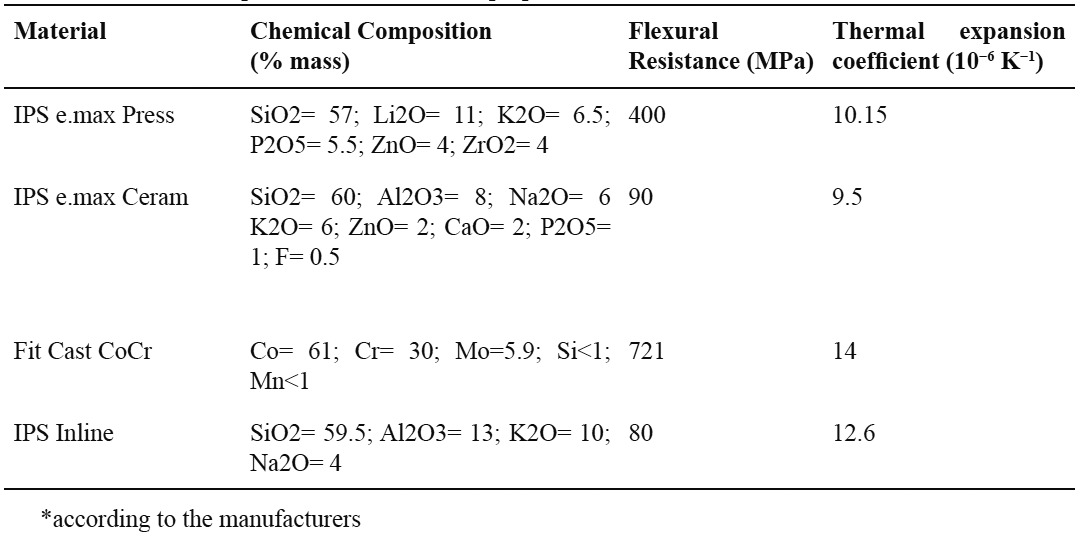
Chemical composition and mechanical properties of the materials tested.

A cylindrical stainless steel matrix with 30mm diameter and 20mm length with a central perforation with 6.5mm depth and 6mm diameter was used to make the specimens and to perform shear strength tests as described by Scollaro et al. (4).

Ten cylindrical shape infrastructures were made per group (n=10) with a 2mm depth spacer disc inserted in the central perforation of the matrix resulting in cylindrical wax patterns with 4.5mm thickness and 6mm diameter. To the CoCr group, it was followed the recommendations of manufacturer to the inclusion, casting and divesting procedures. To the LD groups it was followed the recommendations of manufacturer to inclusion, injection and divesting procedures. However, to LD-untreated group, it was not performed the treatment with acid solution composed by approximately 0.6% of hydrofluoric acid and 1.7% of sulfuric acid during 20 minutes after divesting, as recommended by the manufacturer. The porcelain was applied according to the instructions of manufacturer to prepare masses, condensation, temperature and burning time. To the application, it was used the same matrix described previously, however, without the space disc in position, in order to achieve a porcelain cylindrical shape with 6mm of diameter and 2mm of thickness. Additional LD specimens with no porcelain applied were made for the surface characterization.

The specimens were adapted to the matrix with the spacer disk in position in order to maintain only the porcelain portion outside to apply the force on the infrastructure/porcelain interface. The tests were performed in universal testing machine (Dinamômetros Kratos, São Paulo – SP, Brazil). The force was applied with a loading cell of 100kgf with velocity of 0.5mm/min. The values of bond strength were calculated in MPa dividing the registered force by the sectional area of the specimen.

- Fracture analysis

Fractured surfaces were analyzed in a stereomicroscope (Stemi 2000-C Karl Zeiss). All the samples were analyzed in their infrastructure portion and in the veneering porcelain portion. Fractures were classified as A: Adhesive; CP: Cohesive in the veneering porcelain; CI: Cohesive in the infrastructure and M: Mixed.

- Surface morphology analysis

The analysis performed in scanning electronic microscope (JSM-6380-LV,JEOL, Tokyo, Japan) allowed a visualization of the morphological differences obtained before and after the treatment with acid solutions. The fractured samples were cleaned using Ultrasound apparatus (Vitasonic II, Vita Zanhfabrik) with high frequency (35 kHz), immersed in isopropyl alcohol during 10 minutes and then complete dried. The metallization was performed with a thin conductive layer in gold (50 a 100 Angstrom). The images were obtained by scanning of secondary beam of electrons using high vacuum with accelerating voltage of 15kV.

- Statistical analysis

The statistical analysis was performed using a software (STATISTICA version 5.5, StatSoft Inc., 2000). The results were analyzed by 1 way ANOVA with a level of significance of 5%. Multiple comparisons were performed using Tukey’s test.

## Results

- Shear bond strength

The control group (CoCr) showed significant highest bond strength values and there were no differences between groups LD-treated and LD-untreated.  Table 2 shows the results of 1-way ANOVA analysis. The means, standard deviations and multiple comparisons (Tukey’s Test) are showed in  Table 3.

**Table 2:**
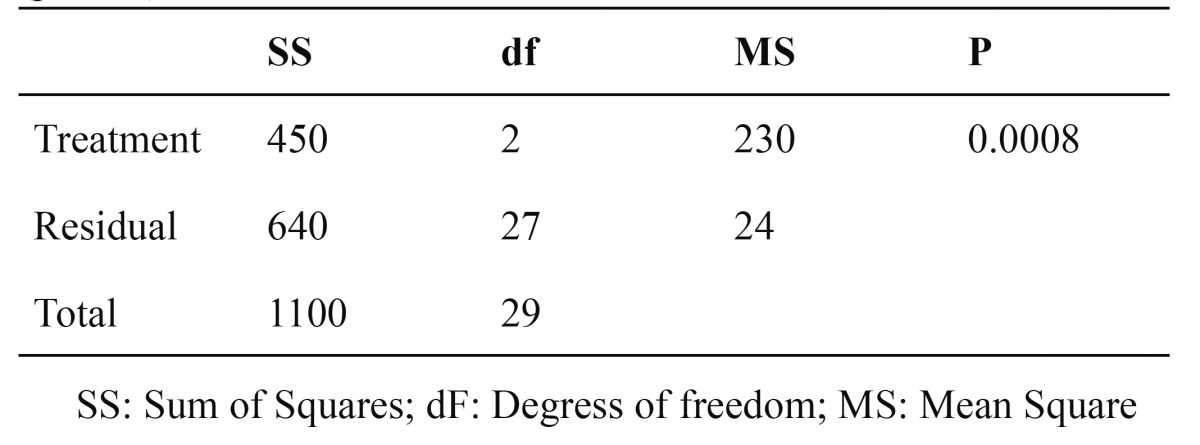
Results of one-way ANOVA for shear bond strength data (p<0.05).

**Table 3:**
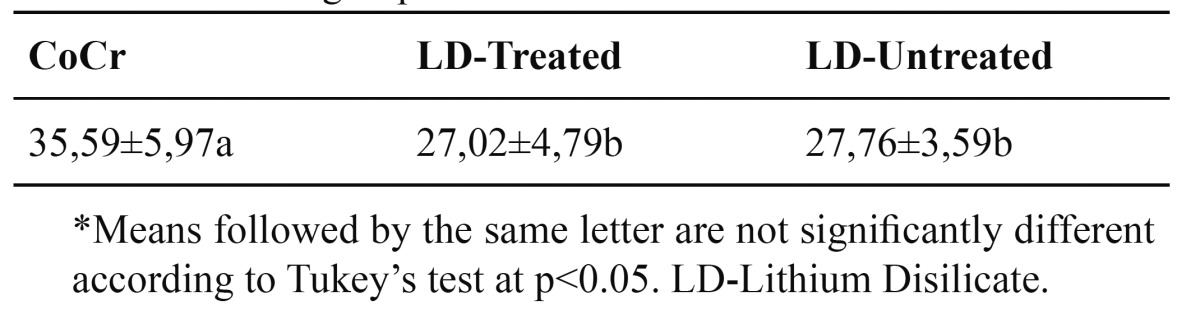
Means, standard deviations and multiple comparisons between the different groups tested.

- Fracture analysis

**Figure 1:**
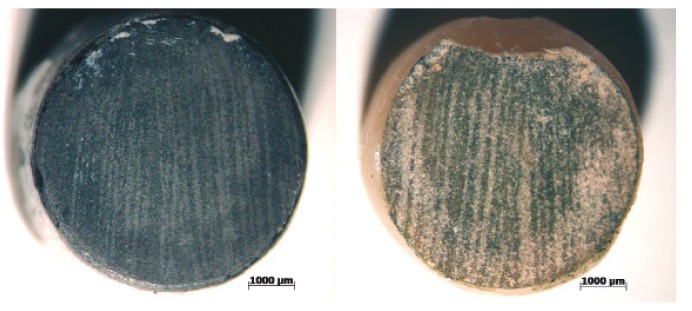
Representative image of fracture for the control group (CoCr) visualized in optical microscopy. Infrastructure portion is on the left and veneering porcelain portion is on the right. Observe the oxide layer adhered to the porcelain.

**Figure 2:**
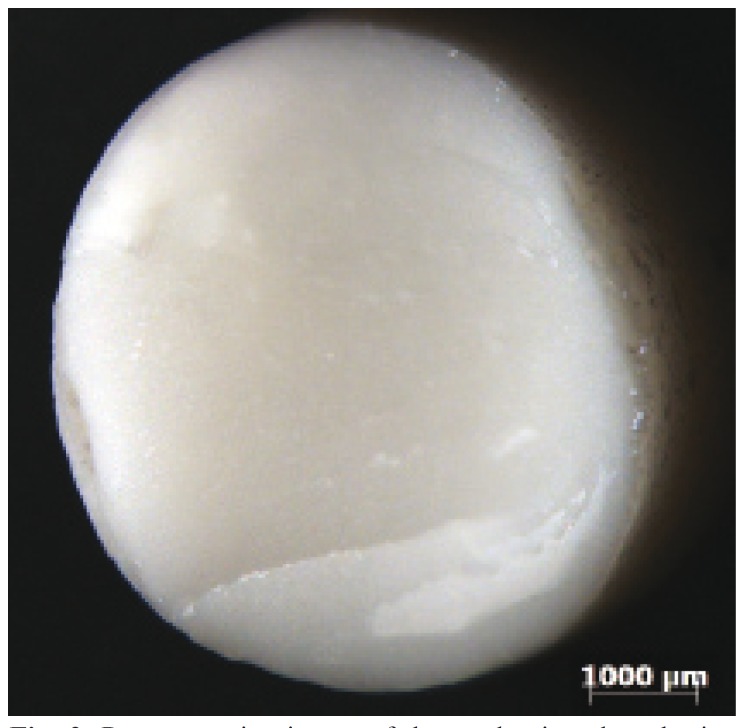
Representative image of the predominantly cohesive fracture of the infrastructure for the lithium disilicate (LD) groups visualized under optical microscopy.

Distinct failures among the groups were found. The control group (CoCr) showed predominantly adhesive failures and the fragments of veneering porcelain took away the oxide layer, suggesting adhesive failure between the oxide layer and the metal. The groups LD-treated and LD-untreated presented predominantly cohesive failure in the infrastructure. Representative images of the fractures can be observed below (Fig. 1,2). The failure modes can be visualized on  Table 4.

**Table 4:**
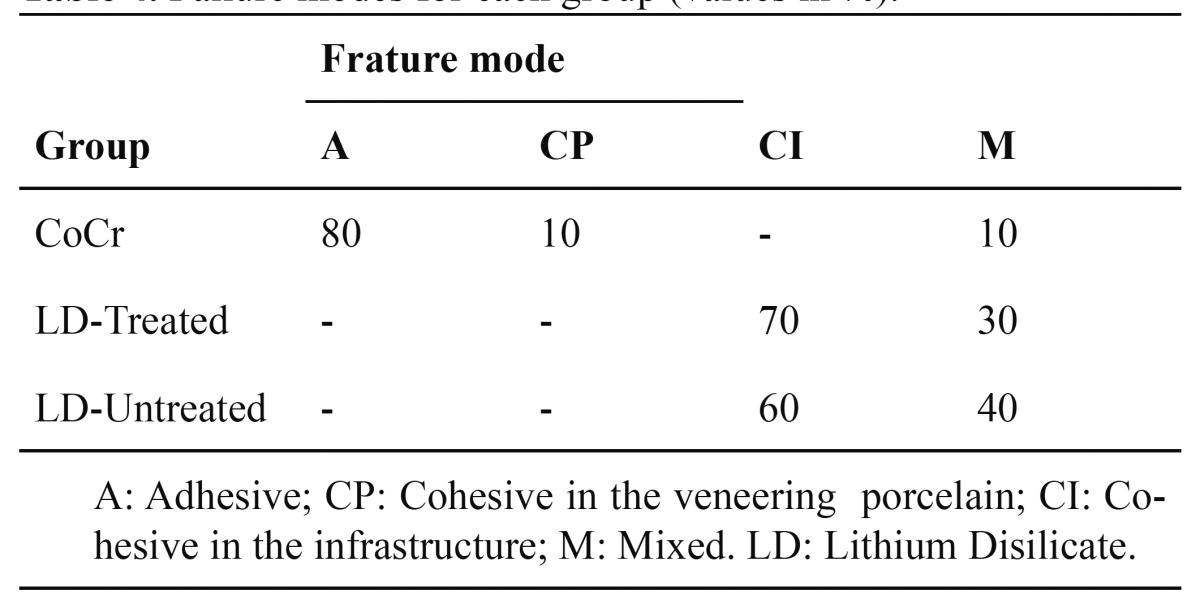
Failure modes for each group (values in %).

- Morphological analysis of lithium disilicate the surfaces 

**Figure 3:**
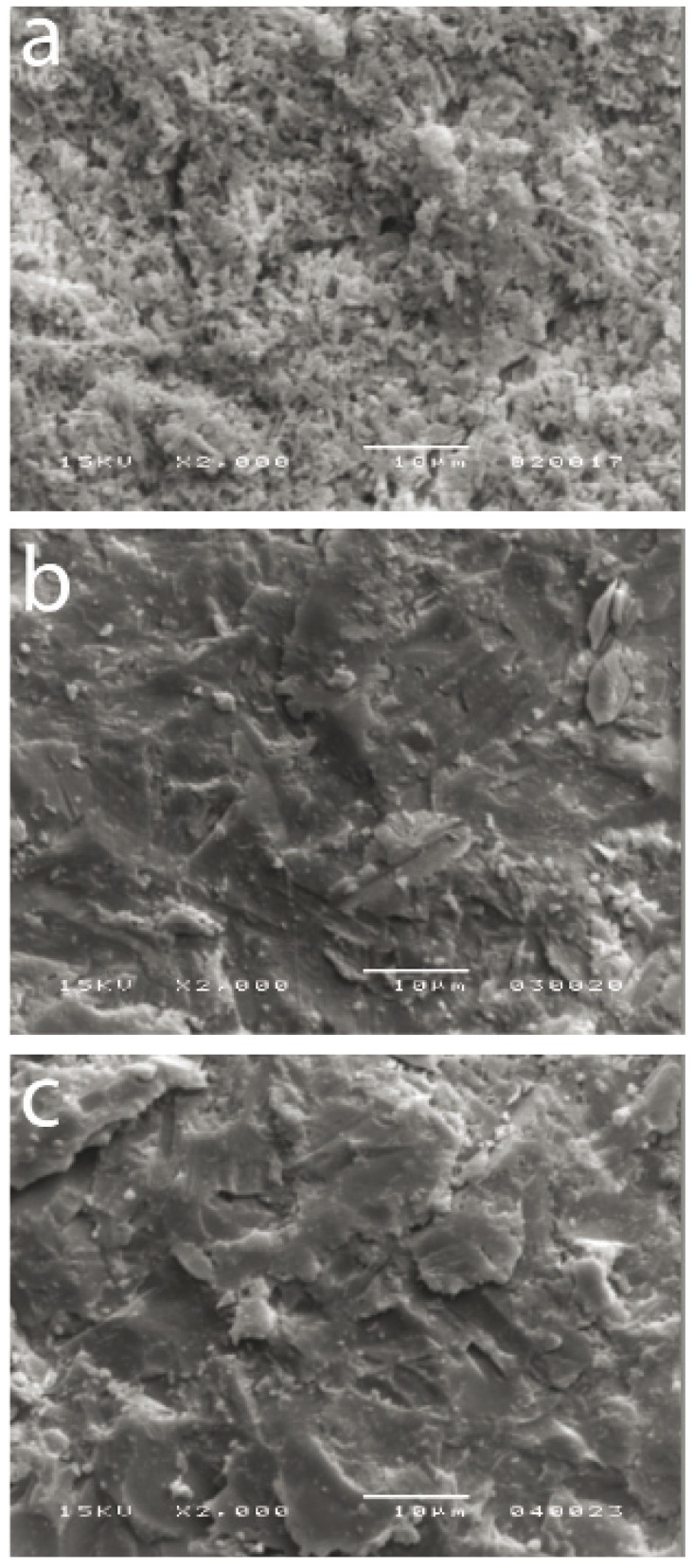
A-Lithium disilicate surface of after treatment with 10% hydrofluoric acid during 20 seconds; B- Lithium disilicate surface after treatment with low concentration acid solution during 20 minutes; C- Lithium disilicate surface without treatment.

The lithium disilicate surfaces were analyzed after different treatments. On the 2000x magnifying it was observed that, when the 10% hydrofluoric acid was applied during 20 seconds, there was the dissolution of the glassy phase, exposing the lithium disilicate crystals (Fig. 3). When the acid solution composed by approximately 0.6% hydrofluoric acid and 1.7% sulfuric acid was applied, the image was very similar with the absence of treatment, indicating that this solution was ineffective to modify surface morphology (Fig. 3).

## Discussion

The interfaces between veneering porcelain and metallic infrastructure are a recurrent theme in the evolution of metal mechanic systems. Likewise, these interfaces play an important role in the mechanical behavior of all ceramics restorations. The stability of those interfaces depends on some factors, such as the wetting capacity of the veneering porcelain, the presence of micromechanical retentions, the chemical bonding and the thermal compatibility between the materials (13).

In this study, the control group CoCr showed 35.59±5.97 MPa of bond strength, satisfactory value for the strength of the bond of metal mechanic systems according to the International Organization for Standardization. The fractures were predominantly adhesives. Similar findings for CoCr alloy were published by Pretti et al. (14) and de Melo et al. (15).

The group LD-treated showed 27.02±4.79 MPa and no significant difference compared to the group LD-untreated (27,76±3,59 MPa). These findings are similar to the Ereifej et al. (11), that found, following the recommendations of the manufacturer, 28.8 MPa of shear strength and also fractures predominantly cohesive in the infrastructure fracture for the IPS e.max Press system. It was suggested that there is a good bonding between the veneering porcelain and the lithium disilicate infrastructure. This can be explained by the similarity in the chemical composition of the IPS e.max Ceram porcelain and the lithium disilicate (IPS e.max Press), mainly related to SiO2 e a o K2O. The IPS e.max Ceram porcelain contains 60% in mass of SiO2, if compared to the 57% of lithium disilicate, and 6% of K2O, when compared to the 6.5 % of lithium disilicate, what would provide a chemical bond between the materials in the firing process of the veneering porcelain (11).

In this study, the application of the acid solution composed by 0.6% hydrofluoric acid and 1.7% sulfuric acid did not have influence on the shear bond strength of lithium disilicate/veneering porcelain interface, what indicates that the acid treatment does not improve the bonding between the materials tested.

The shear bond strength test is widely used to analyze the interfaces between veneering porcelain and metal and all ceramic materials (16). The classical definition of shear tension is the tendency to the sliding of a portion of a body over the other. However, the shear strength tests usually do not cause only the shear tension on the tested specimens. Van Noort et al. (17) demonstrated, by finite element analysis, that the tension distribution can be variable along the interface or even within the veneering and/or infrastructure material. Because of that, fracture analysis is imperative for the interpretation of the results. In the present study, the failure modes for both LD groups were very similar, as evidenced in  Table 4. Therefore, the experimental condition did not influence on the fracture pattern of the tested specimens.

Lithium disilicate is an acid sensitive material. The acid treatment with 10% hydrofluoric acid causes morphological changes in the surface, as observed on Fig. 3, and is responsible for an improvement of the microme-chanical retention of the materials such as resinous cements (7). However, the Scanning Electron Microscopy (SEM) images obtained during this study, showed that the treatment with acid solution composed by 0.6% hydrofluoric acid and 1.7% sulfuric acid did not provided significant morphological differences in the surface of the material (Fig. 3) when compared to no treatment at all (Fig. 3), which suggests, combined with shear bond strength data, that it is not essential to achieve good bonding between the materials.

This study evaluated the influence of low concentration acid treatment in the bond strength between lithium disilicate/veneering porcelain; no significant differences were found in the shear bond strength test, nor in the surface morphology analyzed by SEM. However, because of the limitation of the analysis, it is not possible conclude that this treatment can be dispensable in the laboratorial practice. In this study, the tests performed evaluated only the bond strength of the materials in static load. Studies involving mechanical fatigue and thermal challenges should be performed to enlighten the importance of this procedure in the performance of the lithium disilicate/veneering porcelain interface.
